# Oesophageal varices, schistosomiasis, and mortality among patients admitted with haematemesis in Mwanza, Tanzania: a prospective cohort study

**DOI:** 10.1186/1471-2334-14-303

**Published:** 2014-06-03

**Authors:** Awilly A Chofle, Hyasinta Jaka, Mheta Koy, Luke R Smart, Rodrick Kabangila, Fiona M Ewings, Humphrey D Mazigo, Warren D Johnson, Daniel W Fitzgerald, Robert N Peck, Jennifer A Downs

**Affiliations:** 1 Department of Internal Medicine, Bugando Medical Centre, Box 1370, Mwanza, Tanzania; 2 Department of Internal Medicine, Catholic University of Health and Allied Sciences-Bugando, Mwanza, Tanzania; 3 Department of Medicine, Weill Cornell Medical College, New York, USA; 4 Department of Infectious Disease Epidemiology, London School of Hygiene and Tropical Medicine, London, United Kingdom; 5 Mwanza Interventional Trials Unit, Mwanza, Tanzania; 6 Department of Parasitology, Catholic University of Health and Allied Sciences - Bugando, Mwanza, Tanzania

**Keywords:** Upper gastrointestinal bleeding, Schistosomiasis, Tanzania, Mortality, Prospective, Ultrasound

## Abstract

**Background:**

Upper gastrointestinal bleeding (UGIB) is a common cause of hospital admissions worldwide. Aetiologies vary by sociodemographics and geography. Retrospective studies of endoscopies in much of Africa have documented oesophageal varices as a leading cause of UGIB. Prospective studies describing outcomes and associations with clinical factors are lacking.

**Methods:**

We conducted a prospective cohort study at a referral hospital in Mwanza, Tanzania where schistosomiasis is endemic. Adults admitted with haematemesis underwent laboratory workup, schistosomiasis antigen testing and elective endoscopy, and were followed for two months for death or re-bleeding. We assessed predictors of endoscopic findings using logistic regression models, and determined prediction rules that maximised sensitivity and positive predictive value (PPV).

**Results:**

Of 124 enrolled patients, 13 died within two months (10%); active schistosomiasis prevalence was 48%. 64/91(70%) patients had oesophageal varices. We found strong associations between varices and numerous demographic or clinical findings, permitting construction of simple, high-fidelity prediction rules for oesophageal varices applicable even in rural settings. Portal vein diameter ≥ 13 mm or water sourced from the lake yielded sensitivity, specificity, PPV and NPV > 90% for oesophageal varices; presence of splenomegaly or water sourced from the lake maintained sensitivity and PPV > 90%.

**Conclusions:**

Our results guide identification of patients, via ultrasound and clinical examination, likely to have varices for whom referral for endoscopy may be life-saving. Furthermore, they support empiric anti-schistosome treatment for patients with UGIB in schistosome-endemic regions. These interventions have potential to reduce UGIB-related morbidity and mortality in Africa.

## Background

Upper gastrointestinal bleeding (UGIB), defined as intraluminal bleeding from any location between the upper oesophagus to the duodenum at the ligament of Treitz [1,2], remains a common cause of hospitalisation and death among adults worldwide [3]. In the US, UGIB is responsible for more than 300,000 hospital admissions annually, with a mortality rate of 7-10% [4]. At our hospital in Tanzania, a diagnosis of schistosomiasis or cirrhosis, which was associated with UGIB in more than 2/3 of patients, was recently documented to be the fourth leading cause of both admissions and deaths in the medicine wards [5].

UGIB can be categorised as oesophageal variceal and non-variceal bleeding. Underlying aetiologies of UGIB vary markedly with geographic region and socioeconomic status [6]. Determination of the aetiology of UGIB is essential for optimising management of these critically-ill patients. Oesophageal band ligation for patients with bleeding oesophageal varices is the ideal treatment that has been shown to reduce mortality [7]. In addition, if the underlying cause of the varices can be determined and is treatable (including infectious aetiologies such as schistosomiasis), pathology leading to oesophageal varices may be diminished.

Two prior retrospective studies in Tanzania, including one at our own hospital, have documented a startlingly high prevalence of variceal bleeding among adults with UGIB [8,9]. In these two studies among adults with UGIB who underwent endoscopy, oesophageal varices were present in 51% and 42% of patients. These rates contrast with those from other parts of the world in which the prevalence of variceal bleeding among those with UGIB is < 10% [10,11]. Less common aetiologies of UGIB in the Tanzanian studies included duodenal ulcers (14% and 15%), gastric ulcers (11% and 5%) and gastritis (13% and 8%). Of note, both Tanzanian studies were retrospective reviews and therefore did not characterise all patients admitted with UGIB, but only those who were able to afford and undergo endoscopy. In addition, these studies were limited to analysis of data that had been recorded by clinicians in patients’ charts.

In this prospective cohort study, we sought to determine aetiologies of UGIB among adults admitted to our hospital and demographic and clinical factors associated with oesophageal varices that would be useful for guiding the diagnosis and management of patients presenting with UGIB to African hospitals where endoscopy is unavailable or unaffordable. We hypothesised that the prevalence of oesophageal varices in this region in which schistosomiasis is hyper-endemic would be comparable to or higher than the prevalence found in prior retrospective studies. We further hypothesised that ongoing active schistosomiasis would a frequent finding and would be associated with oesophageal varices.

## Methods

### Study setting

We aimed to enroll all adult inpatients presenting with haematemesis to our hospital, Bugando Medical Centre (BMC), over a period of six months. BMC is a regional hospital that serves the Lake Zone of north-western Tanzania (population of ~13 million) and is located in the city of Mwanza. Schistosomiasis is highly endemic in the region, with a documented prevalence of > 50% in many areas [12-14]. The regional prevalence of chronic Hepatitis B infection is ~10% [15]. All adults (≥ 14 years) who reported haematemesis in the past 14 days were interviewed for enrolment. Enrolled patients were followed for two months after the date of admission for death or rebleeding, the time period during which mortality risk is highest [16]. Telephone contact with patients or family members was used to determine outcomes among discharged patients. Rebleeding was defined as recurrent vomiting of fresh blood either with shock or a decrease in haemoglobin concentration of > 2 g/dL.

### Data collection

Patients were interviewed and examined within 24 hours of admission using a structured questionnaire to collect demographic information, clinical symptoms and physical signs. Initial management of all patients with UGIB at our hospital includes the following: evaluation of cardiovascular status using pulse, blood pressure, and orthostatic changes, confirmation of adequate intravenous access, rapid intravascular volume replacement with fluids and red blood cells, and correction of coagulopathy with platelet concentrate or fresh frozen plasma. For all study patients, ten millilitres of blood were collected for cross-matching and to measure haemoglobin level, platelets, mean corpuscular volume (MCV), international normalised ratio (INR), partial thromboplastin time (PTT), rapid test for HIV, hepatitis B surface antigen (HBsAg), hepatitis C antibody (HCAb), alanine aminotransferase (ALT), aspartate aminotransferase (AST), total and direct bilirubin, and albumin. Decisions regarding blood transfusion were made by the attending physicians and depended on estimated blood loss, the availability of blood at the blood bank, and the patient’s haemoglobin level.

Each patient’s urine was tested for circulating cathodic antigen (CCA), an antigen produced by adult schistosome worms that is secreted into the bloodstream and excreted into urine, using a point-of-care test (Rapid Medical Diagnostics, Pretoria, South Africa). The CCA test indicates active schistosome infection and can be positive in the urine during infection with either species of schistosomes that are endemic in Tanzania (*S. mansoni* and *S. haematobium*), though its sensitivity is lower in *S. haematobium*[17-19]*.* CCA point-of-care testing is used widely and has been found to be more sensitive than the gold standard Kato-Katz stool diagnosis of *Schistosoma mansoni,* particularly for lighter infections [20]. Following the manufacturer’s instructions, any positive line in the “test” area was considered positive. Line intensities were graded as “1” (test line visible but lighter than control line), “2” (test line equal to control line), and “3” (test line darker than control line).

Endoscopy was performed using a Pentax EPM 3500 fiberoptic endoscope (Pentax Medical, Tokyo, Japan) for patients who were able to afford endoscopy (~100 US dollars). Oesophageal banding was performed for patients who had bleeding varices. Injection with diluted adrenaline was performed for bleeding peptic ulcers.

We also asked patients ten questions derived from Oxford University’s Multidimensional Poverty Index (MPI), which is an international measure of poverty relevant to 109 resource-poor countries [21]. The MPI is designed to measure deprivations across the three dimensions of education, health, and standard of living, and persons reporting deficiencies in at least one-third of the measures are considered multidimensionally poor. The questionnaire is scored from zero to six, with six indicating no poverty and scores less than or equal to four indicating multidimensional poverty (Additional file 1: Table S1).

### Data analysis

Data were entered into Microsoft Excel and analysed with Stata/IC Version 12 (College Station, Texas). Categorical variables were summarised by frequency and percentage, and continuous variables by median and interquartile range (IQR). Statistical tests between categorical dependent and independent variables were done using Chi-squared or Fisher’s exact tests. We assessed demographic and clinical variables for associations with oesophageal varices using exploratory univariable logistic regression models. Age was categorised as 14–29, 30–44, 45–59 and ≥ 60 years. Occupation and water source were collapsed to binary variables indicating fisherman and water sourced from the lake, respectively. Continuous variables were assumed to have linear effects, and were checked for departures from linearity by categorising and assessing using Wald tests. We also examined continuous variables as binary (normal/abnormal for standard accepted medical values) to facilitate the generation of prediction rules. We created possible prediction rules for oesophageal varices using binary variables which reached p < 0.05 and which were immediately ascertainable at the bedside (that is, those requiring laboratory testing were omitted; age was also omitted since there was not a clear linear relationship). We generated rules based on all combinations of these binary variables, using "or" operators. Rules were categorised as clinical only or requiring ultrasound (to measure portal vein diameter). We specified a priori that adequate rules would have sensitivity and positive predictive value (PPV) ≥ 90%, with highest priority given to sensitivity. Rules with specificity and negative predictive value (NPV) ≥ 85% were preferred. For the sake of parsimony, additional variables were only added to the prediction rules if they improved test characteristics. 95% confidence intervals (CIs) were estimated using Wilson's formula.

### Ethical issues

Ethical approval was obtained from Bugando Medical Centre and Weill Cornell Medical College. Patients or legal surrogates provided written informed consent for study participation.

## Results

### Study population

Between 1 June and 31 December 2012, 132 adults presented to Bugando with haematemesis. Of these, four died before they could be enrolled, two did not consent for study participation, and two were admitted directly to the Department of Surgery. The remaining 124/132 (94%) patients were enrolled in our study.

Demographic and medical history information of these 124 patients are presented in Table 1. Patients’ median age was 38 (IQR 30–52) years, and the majority (60%) were male. Many patients were peasants (36%), Fishermen (14%) or otherwise self-employed (38%), and most had received only primary education or less (83%). Over a third of patients (35%) met the criteria for multidimensional poverty. Alcohol use in the past week was reported in 27 (22%). Fifty patients (40%) had been treated with praziquantel previously, while only 47 (38%) had access to piped water.

**Table 1:** Demographic Characteristics of 124 Patients Admitted to Bugando Medical Centre with Upper Gastrointestinal Bleeding

**Variable**	**Response**	**N = 124**	**%**
Male sex		75	60%
Age group (years)	14-29	30	24%
	30-44	52	42%
	45-59	20	16%
	≥ 60	22	18%
Live within 5 km of the lake	Yes	80	65%
Current occupation	Peasant	45	36%
	Fisherman	17	14%
	Other self-employed	47	38%
	Civil servant	9	7%
	Student	6	5%
Highest education obtained	No or less than primary	26	21%
	Completed primary education	77	62%
	Completed secondary education	8	6%
	Any college/university education	13	10%
Source of water	Piped	47	38%
	Lake	41	33%
	River	26	21%
	Other (rain water, wells, dams)	10	8%
Poverty index*	≤ 2	7	6%
	2.1-4.0	36	29%
	> 4.0	81	65%
Alcohol use in the past week	Yes	27	22%
Aspirin or NSAID use in the past week	Yes	2	2%
Reported history of prior haematemesis	Yes	47	38%
Reported history of prior praziquantel treatment	Yes	50	40%

*Poverty index > 4 indicates that a person does not have multidimensional poverty.

### Physical and laboratory findings

Notable physical findings at presentation included the following: splenomegaly in 83 (67%), ascites in 47 (38%), abnormally small liver span (< 8 cm) in 24 (19%), haemodynamic instability in 26 (21%), hepatomegaly (≥ 13 cm) in 18 (15%), and new or worsening encephalopathy in four (3%). Ultrasound examination confirmed the physical examination finding of splenomegaly in 81/83 cases (97.5%) and the remaining two cases were borderline by ultrasonographic measurement.

Median admission haemoglobin level was 4.6 (3.8-6.9) g/dL. Sixty-seven (54%) patients had mean corpuscular volumes < 76 fl, suggesting chronic blood loss. Forty-six (37%) patients had abnormal INRs > 1.5, and 40 (32%) had an elevated total bilirubin level > 17 μmol/L. Transaminases were elevated > 40 IU/L in 33 (27%). The urine CCA test was positive in 59 patients (48%). Of these, it was graded as one in 38 patients, two in 14 patients, and three in seven patients.

### Outcomes

Thirteen of 124 enrolled patients died (10%): nine (69%) during the first week, two (15%) during the second week and two (15%) during the third and fourth weeks, after they had been discharged. Sixty-two patients (50%) experienced rebleeding within two months of admission. Of these, 53 (85%) had in-hospital rebleeding, and the remaining nine (15%) had rebleeding that necessitated readmission. Of note, four had severe rebleeding, all from bleeding peptic ulcer disease, and all four underwent surgery. The median transfused units was three (IQR 1–4 units).

### Endoscopic findings

**Figure 1:**
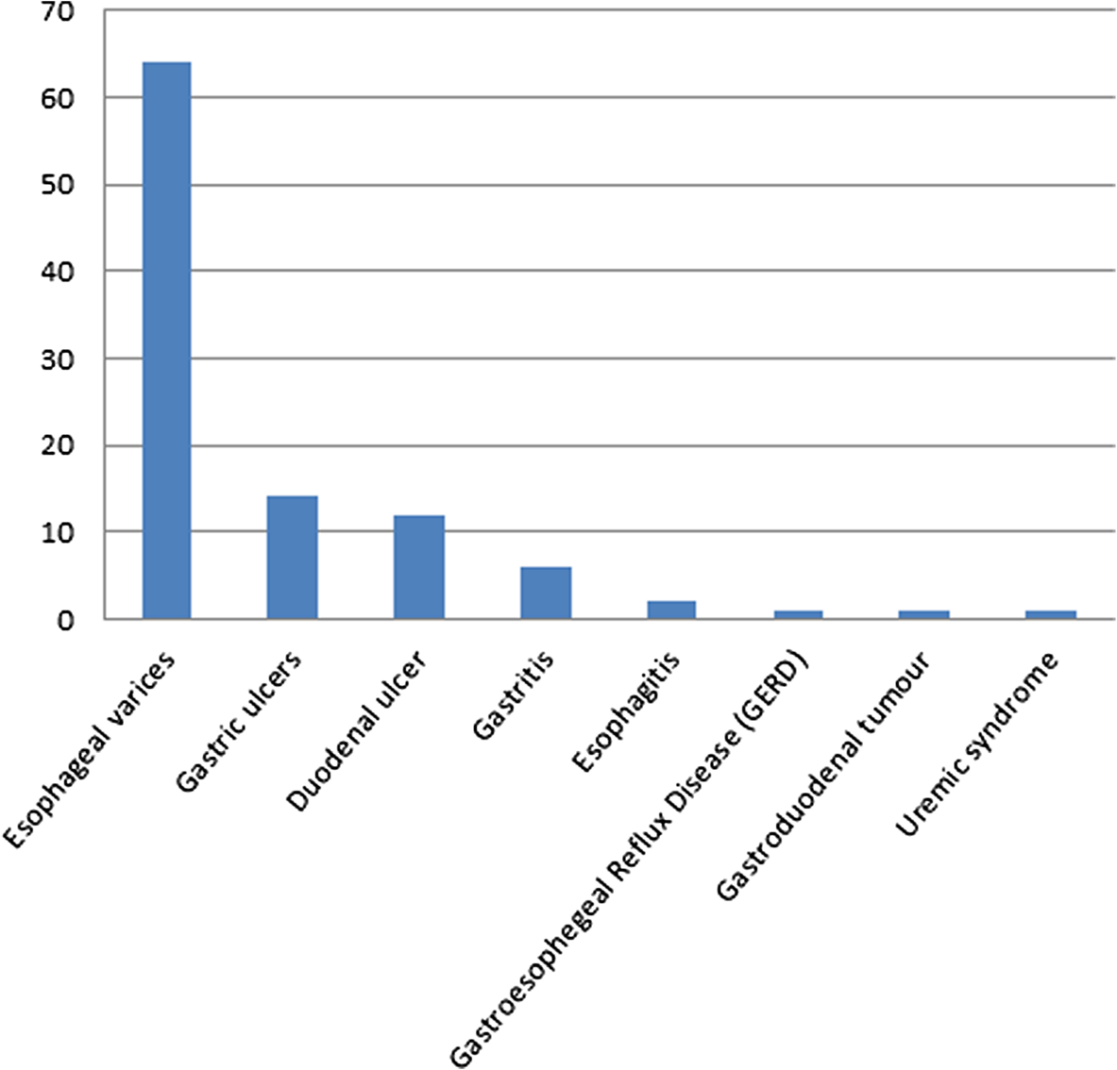
Endoscopically- and/or medically-confirmed aetiologies of upper gastrointestinal bleeding among 91 patients admitted to Bugando Medical Centre.

Of 124 patients enrolled in the study, 91 (73%) underwent endoscopy and received aetiological diagnoses for their haematemesis (Figure 1). Endoscopy was performed at a median of two (IQR 2–5) days. Oesophageal varices were the most common lesions found, affecting 64 (70%) of patients who underwent endoscopy. The next most common diagnoses were gastric and duodenal ulcers (14 (15%) and 12 (13%), respectively).

Among the 13 deaths, seven were in the group of 33 patients who did not have endoscopy, and six were in the 91 patients who did have endoscopy (21% versus 7%, respectively, p = 0.02). The odds ratio for death was 3.8 (95% CI 1.2-12.4; p = 0.03) for those who did not versus did undergo endoscopy.

### Predictors of oesophageal varices

Factors significantly associated with variceal bleeding on univariable logistic regression analysis included younger age, living within five kilometres of the lake, working as a fisherman, hepatitis B antigen positivity, schistosome CCA positivity, history of praziquantel treatment, lower blood pressure, ascites, splenomegaly, larger portal vein diameter, lower haemoglobin level, lower mean corpuscular volume, and elevated total bilirubin (Table 2). Given the small sample size and some strong predictors of variceal bleeding, it was not possible reliably to estimate multivariable models. For example, the diagnosis for all fishermen was variceal bleeding, and fitting even just bivariable models with factors such as age and portal vein diameter resulted in hugely inflated odds ratios.

**Table 2:** Demographic and Clinical Predictors of Oesophageal Varices among 91 Patients with Endoscopic Diagnoses

**Demographic or clinical parameter**	**Patients without varices (N = 27) median (IQR) or number (%)**	**Patients with varices (N = 64) median (IQR) or number (%)**	Univariable odds ratio [95% CI]*	P-value*
Sex				0.88
Female	11 (41%)	25 (39%)	1 [reference]	
Male	16 (59%)	39 (61%)	1.07 [0.43-2.68]	
Age, years				0.002
15-29	6 (22%)	11 (17%)	1 [reference]	
30-44	6 (22%)	35 (55%)	3.18 [0.85-11.9]	
45-59	3 (11%)	12 (19%)	2.18 [0.44-10.9]	
≥ 60	12 (44%)	6 (9%)	0.27 [0.07-1.10]	
Live within 5 km of the lake				**< 0.001**
No	19 (70%)	11 (17%)	1 [reference]	
Yes	8 (30%)	53 (83%)	11.4 [4.00-32.7]	
Working as a fisherman				0.002***
No	27 (100%)	48 (75%)	***	
Yes	0	16 (25%)		
Highest education obtained				0.10
No or less than primary	8 (30%)	11 (17%)	1 [reference]	
Completed primary	12 (44%)	45 (70%)	2.73 [0.90-8.29]	
Completed secondary	1 (4%)	3 (5%)	2.18 [0.19-25.0]	
Any college/university	6 (22%)	5 (8%)	0.61 [0.14-2.71]	
Source of water				< 0.001***
Piped/river/other	27 (100%)	31 (48%)	***	
Lake	0	33 (52%)		
Poverty index**				0.61
≤ 4	7 (26%)	20 (31%)	1 [reference]	
> 4	20 (74%)	44 (69%)	0.77 [0.28-2.11]	
Alcohol use in the past week				0.19
No	19 (70%)	53 (83%)	1 [reference]	
Yes	8 (30%)	11 (17%)	0.49 [0.17-1.41]	
Aspirin/NSAID use in the past week				0.54
No	26 (96%)	62 (98%)	1 [reference]	
Yes	1 (4%)	1 (2%)	0.41 [0.02-6.85]	
**Prior medical history**				
Reported history of prior haematemesis				0.06
No	21 (78%)	36 (56%)	1 [reference]	
Yes	6 (22%)	28 (44%)	2.72 [0.97-7.65]	
Reported history of prior praziquantel use				**0.008**
No	22 (81%)	32 (50%)	1 [reference]	
Yes	5 (19%)	32 (50%)	4.40 [1.48-13.1]	
**Clinical examination**				
Mean arterial blood pressure (mmHg)	83 (70–87)	70 (63–82)	0.67 [0.49-0.93]+	**0.02**
Heart rate (beats per minute)	98 (88–110)	105 (86–111)	1.00 [0.98-1.03]	**0.78**
Ascites				**0.002**
No	24 (89%)	32 (50%)	1 [reference]	
Yes	3 (11%)	32 (50%)	8.00 [2.18-29.2]	
Hepatomegaly				0.98
No	24 (89%)	57 (89%)	1 [reference]	
Yes	3 (11%)	7 (11%)	0.92 [0.23-4.12]	
Splenomegaly				**< 0.001**
No	23 (85%)	5 (8%)	1 [reference]	
Yes	4 (15%)	59 (92%)	67.9 [16.7-275]	
Encephalopathy				0.54
No	26 (96%)	63 (98%)	1 [reference]	
Yes	1 (4%)	1 (2%)	0.41 [0.02-6.85]	
**Investigations**				
Hepatitis B surface Ag				**0.03**
Negative	25 (93%)	45 (70%)	1 [reference]	
Positive	2 (7%)	19 (30%)	5.27 [1.14 – 24.5]	
Anti-Hepatitis C Ab				0.54
Negative	26 (96%)	63 (98%)	1 [reference]	
Positive	1 (4%)	1 (2%)	0.41 [0.02-6.85]	
HIV				0.63
Negative	26 (96%)	60 (94%)	1 [reference]	
Positive	1 (4%)	4 (6%)	1.73 [0.18 – 16.3]	
Schistosoma CCA				**0.01**
Negative	19 (70%)	26 (41%)	1 [reference]	
Positive	8 (30%)	38 (59%)	3.47 [1.32-9.11]	
Portal vein, mm				**< 0.001**
< 13	25 (93%)	3 (5%)	1 [reference]	
≥ 13	2 (7%)	61 (95%)	254 [40.0-1614]	
Haemoglobin (g/dL)	6.0 (4.0-9.9)	4.2 (3.6-5.6)	0.76 [0.64-0.91]	**0.002**
MCV (fl)	82 (71–89)	74 (67–80)	0.59 [0.37-0.92]+	**0.02**
Platelets (10^9^/L)	150 (103–215)	103 (55–143)	0.70 [0.45-1.11]++	0.13
PTT (seconds)	28 (26–32)	30 (27–35)	1.69 [0.75-3.79]+	0.20
INR (ratio)	1.2 (1.0-1.4)	1.4 (1.1-1.7)	1.37 [0.51-3.63]	0.53
Total bilirubin (μmol/L)	8.8 (5.2-12.7)	13.2 (7.7-21.7)	1.07 [1.01-1.16]	**0.04**
Albumin (g/L)	35 (30–44)	32 (28–39)	0.74 [0.44-1.24]+	0.25
AST (U/L)	22 (19–29)	28 (19–49)	1.23 [0.96-1.57]+	0.10
ALT (U/L)	17 (14–27)	23 (15–47)	1.20 [0.97-1.49]	0.09

*Results from logistic regression models (Wald p-values).

**Poverty index > 4 indicates that a person does not have multidimensional poverty.

***Unable to calculate odds ratio due to a zero; p-value from Fisher's exact test.

+Per 10 units higher.

++Per 100 units higher.

### Prediction rules for oesophageal varices

Seven variables met our criteria for inclusion in the prediction rules: living within 5 km of the lake, fisherman, water sourced from lake, history of praziquantel use, ascites, splenomegaly and portal vein diameter ≥ 13 mm. Considering each alone, splenomegaly and portal vein diameter ≥ 13 mm each met our criteria of sensitivity and PPV ≥ 90% (92 [95% CI 83–97] and 94 [85–98], respectively, for splenomegaly, and 95 [87–98] and 97 [89–99], respectively, for portal vein diameter ≥ 13 mm; Table 3). Portal vein diameter ≥ 13 mm offered greater specificity and NPV (93 [77–98] and 89 [73–96], respectively). Considering combinations of variables, 38 met our criteria. Further restricting to those with specificity and NPV ≥ 85%, only one clinical-only combination remained, namely splenomegaly or water sourced from lake, which yielded some improvement in sensitivity and NPV over splenomegaly alone. Seven further combinations including portal vein diameter ≥ 13 mm remained; the rule based on portal vein diameter ≥ 13 mm or water sourced from lake yielded optimal results (sensitivity 98 [92–100], specificity 93 [77–98], PPV 97 [89–99] and NPV 96 [81–99]).

**Table 3:** Possible Prediction Rules for Oesophageal Varices

**Rule**	**Sensitivity (95% CI)**	**Specificity (95% CI)**	**PPV (95% CI)**	**NPV (95% CI)**
**Single clinical condition:**				
Splenomegaly	92 (83–97)	85 (68–94)	94 (85–98)	82 (64–92)
Live within 5 km of the lake	83 (72–90)	70 (52–84)	87 (76–93)	63 (46–78)
Water sourced from lake	52 (40–63)	100 (88–100)	100 (90–100)	47 (34–59)
Ascites	50 (38–62)	89 (72–96)	91 (78–97)	43 (31–56)
History of praziquantel	50 (38–62)	81 (63–92)	86 (72–94)	41 (29–54)
Fisherman	25 (16–37)	100 (88–100)	100 (81–100)	36 (26–47)
**Ultrasound single condition:**				
Portal vein diameter ≥ 13 mm	95 (87–98)	93 (77–98)	97 (89–99)	89 (73–96)
**Combinations of clinical conditions:**				
Splenomegaly or water sourced from lake	94 (85–98)	85 (68–94)	94 (85–98)	85 (68–94)
**Combinations of clinical and ultrasound conditions:**				
Portal vein diameter ≥ 13 mm or water sourced from lake	98 (92–100)	93 (77–98)	97 (89–99)	96 (81–99)
Portal vein diameter ≥ 13 mm or water sourced from lake or ascites	98 (92–100)	89 (72–96)	95 (87–98)	96 (80–99)
Portal vein diameter ≥ 13 mm or fisherman	95 (87–98)	93 (77–98)	97 (89–99)	89 (73–96)
Portal vein diameter ≥ 13 mm or ascites	95 (87–98)	89 (72–96)	95 (87–98)	89 (72–96)

This table shows single-condition rules and rules which met our primary criteria of sensitivity and PPV ≥ 90%, and additionally had specificity and NPV ≥ 85%.

## Discussion

Our prospective cohort study documents the strikingly high prevalence of variceal bleeding (70%) among 124 consecutive adult inpatients presenting with haematemesis to a regional hospital in western Tanzania. Moreover, we found that nearly 60% of patients presenting with haematemesis due to variceal bleeding and 30% presenting with haematemesis due to other causes had evidence of active schistosomiasis. These results are important for public health in sub-Saharan Africa because they document high rates of a preventable disease. Scaling up efforts to control schistosomiasis could lead to significant reductions in morbidity and mortality among the ~54 million people living in sub-Saharan Africa who have *S. mansoni* infection and are at risk for periportal fibrosis, oesophageal varices, and death [22].

To our knowledge, this is the first prospective study to determine endoscopic diagnoses, presenting features, and outcomes among adults with UGIB in East Africa. Two recent retrospective studies done in Tanzania have documented comparably high rates of oesophageal varices among patients with UGIB (41%-52%) [8,9]. Our study, conducted in a region in which the prevalence of schistosomiasis is one of the highest in the world, additionally describes numerous factors that were highly associated with oesophageal varices in patients presenting with haematemesis. Associated characteristics included demographics, clinical findings at the time of admission, and documented active infections (hepatitis B and schistosomiasis). Taken together, these data suggest that patients who live near the lakeside and/or fished and present with signs of portal hypertension and liver dysfunction are highly likely to have variceal bleeding.

Identification and appropriate management of patients who have oesophageal varices is urgent because each episode of variceal haemorrhage carries 18-30% risk of death within six weeks with increasing mortality in the ensuing months and years [23-25]. These mortality estimates are from wealthier settings in which cirrhosis, which is associated with higher mortality, is more common but additional management options such as sclerotherapy and portosystemic shunting are available. We believe that mortality in resource-limited settings, particularly when patients often present with more advanced disease or live remotely from advanced medical care, may be higher. Moreover, endoscopy is a scarce resource in Tanzania, and when combined with transportation expenses, it easily costs over a month’s wages for an average rural patient. Therefore, identification of patients most likely to benefit from endoscopy (e.g., those with oesophageal varices that can be banded or sclerosed) is additionally important from a healthcare cost and resource utilisation perspective.

We determined clinical prediction rules with high sensitivity, specificity, PPV, and NPV that can be used in smaller hospitals where ultrasounds but not endoscopy are available, and in even smaller clinics using history and physical examination alone. When ultrasound is available, the single finding of portal vein diameter ≥ 13 mm has over 90% sensitivity, specificity and PPV for oesophageal varices. When ultrasound is not available, the presence of splenomegaly alone maintains a sensitivity and PPV > 90%, allowing focused use of the scarce resource of endoscopy for patients in whom intervention is possible. These clinical rules therefore represent a powerful tool that can be used to suggest which patients with haematemesis are highly likely to have oesophageal varices. These patients should be referred for urgent endoscopy and counselled that endoscopy may be life-saving.

Our data demonstrates active schistosome infection in nearly half of adults presenting with haematemesis. Similar rates of schistosomiasis in community-based studies of adults in this region have also been documented [12-14]. These results contrast with the common teaching in sub-Saharan Africa that schistosomiasis is predominantly an infection of children and that adults presenting with UGIB due to schistosomiasis have past but not present infection. The prevalence of schistosomiasis in endemic regions peaks between age ten and 20, and adults can develop partial immunity but many still remain infected [26,27]. For this reason, we advocate universal testing and treatment for schistosomiasis and/or empiric anti-schistosome treatment as an integral component of managing patients who present with UGIB in schistosome-endemic settings, particularly given the evidence that schistosomal liver pathology will continue to worsen in the setting of ongoing schistosome infection but may regress following anti-schistosome treatment [28,29].

In addition, our findings suggest that current schistosomiasis control efforts in Tanzania and many other countries in sub-Saharan Africa are inadequate. Many nations with limited healthcare funds focus schistosomiasis treatment efforts on schoolchildren despite the World Health Organization’s recommendations that both children and adults living in highly-endemic areas should be treated annually for schistosomiasis [30]. The average lifespan of the *S. mansoni* worm is 3–5 years, while the time from infection to development of liver fibrosis typically takes 5–15 years [26]. Therefore, the finding of active schistosomiasis in these adults presenting with end-stage liver fibrosis suggests that they have been subject to ongoing exposure and re-infection for many years. In the absence of the ability to eliminate schistosomiasis altogether through behavioural change, clean water provision, and improved sanitation, the next-best remedy is to decrease worm burden and morbidity through routine treatment for both children and adults.

Our study has several limitations. First, we were not able to perform liver biopsies in this resource-limited setting in order to corroborate our diagnoses histopathologically. The ability to do so would have been most useful to determine the predominant disease in the 11 patients who had both active schistosome infection and Hepatitis B antigen positivity. Second, we limited our study population to patients with haematemesis in the past 14 days in order to ensure that only patients with true upper gastrointestinal bleeding were enrolled. It is possible that this led to the exclusion of patients with less acute or lower-volume upper gastrointestinal bleeding.

## Conclusions

In conclusion, our prospective data reveals extremely high rates of oesophageal varices and active schistosomiasis among adults presenting with haematemesis to a referral hospital in a region in which both schistosomiasis and hepatitis B are endemic. Oesophageal varices were strongly associated with specific ultrasonographic and physical examination findings that can be assessed even in the most rural hospitals. Our data provides guidance for identification of patients likely to have varices for whom referral for endoscopy may be life-saving, and it also supports empiric praziquantel treatment for patients in low-resource, schistosome-endemic regions who present with UGIB. Both of these simple interventions have the potential to reduce morbidity and mortality associated with UGIB in sub-Saharan Africa.

## Abbreviations

ALT: Alanine aminotransferase; AST: Aspartate aminotransferase; BMC: Bugando Medical Centre; CCA: Circulating cathodic antigen; HBsAg: Hepatitis B surface antigen; HCAb: Hepatitis C antibody; INR: International normalized ratio; IQR: Interquartile range; MCV: Mean corpuscular volume; MPI: Multidimensional poverty index; NPV: Negative predictive value; PPV: Positive predictive value; PTT: Partial thromboplastin time; UGIB: Upper gastrointestinal bleeding.

## Competing interests

The authors declare that they have no competing interests.

## Authors’ contributions

Designed the study: AC, HJ, MK, HM, RP, JD. Data Collection: AC, HJ, MK. Data Analysis: AC, LS, FE, DF, RP, JD. Data Interpretation: AC, FE, WJ, DF, RP, JD. Manuscript preparation: AC, HJ, MK, LS, RK, FE, HM, WJ, DF, RP, JD. All authors read and approved the final manuscript.

## Pre-publication history

The pre-publication history for this paper can be accessed here:

http://www.biomedcentral.com/1471-2334/14/303/prepub

## Supplementary Material

Additional file 1: Table S1 Supplementary Material: Multidimensional Poverty Index. Click here for file
