# Supplementary material for: Oesophageal varices, schistosomiasis, and mortality among patients admitted with haematemesis in Mwanza, Tanzania: a prospective cohort study
Source: BMC Infect Dis. 2014 Jun 3;14:303. doi: 10.1186/1471-2334-14-303 (PMC4065539; doi:10.1186/1471-2334-14-303)
Supplement: Additional file 1: Table S1 — Supplementary Material: Multidimensional Poverty Index. [file 1471-2334-14-303-S1.pdf]

**Supplementary Material: Multidimensional Poverty Index**

| Question | Answer | How to Score | Points |
|---|---|---|---|
| 1. Of all of the people in your household, has any one person attended school for more than 5 years of his or her life? | 0 – No<br>1 – Yes | 1 point for Yes | |
| 2. How many children between the ages of 5 and 14 are living in your household?<br>How many of these children are currently attending school? | _____ children<br><br>_____ attending school | 1 point if all children attend school. Otherwise 0 points. | |
| 3. Have any children in your household died? | 0 – Yes<br>1 – No | 1 point for No | |
| 4. Is any person in your household too thin (malnourished) because there is not enough food to eat? | 0 – Yes<br>1 – No | 1 point for No | |
| 5. Does your household have electricity, either from Tanesco or from a generator? | 0 – No<br>1 – Yes | 1/3 point for Yes | |
| 6. Does your household have access to safe, clean drinking water? How many minutes does it take you to collect clean water? | 0 – No<br>1 – Yes and it takes less than 30 minutes<br>2 – Yes but it takes longer than 30 minutes | 1/3 point for "Yes and it takes less than 30 minutes"<br><br>Otherwise 0 points. | |
| 7. How many households share the toilet or latrine that you use? | ___ households | 1/3 point for 1-4 households. 0 points if ≥5 households. | |
| 8. Is the floor of your home made from dirt or sand or dung? | 0 – Yes<br>1 – No | 1/3 point for No | |
| 9. What do you use to cook most of the food that you eat at home? | 0 – Wood, coal, or dung<br>1 – Other | 1/3 point for Other | |
| 10. Does your household have the following things:<br>a. Radio<br>b. Television<br>c. Telephone<br>d. Bicycle<br>e. Motorcycle<br>f. Refrigerator<br>g. Car<br>h. Tractor | <br>0 – No / 1 – Yes<br>0 – No / 1 – Yes<br>0 – No / 1 – Yes<br>0 – No / 1 – Yes<br>0 – No / 1 – Yes<br>0 – No / 1 – Yes<br>0 – No / 1 – Yes<br>0 – No / 1 – Yes | <br>0 points if the household has zero or one of the things listed in "a" – "f" and no car or tractor.<br><br>Otherwise 1/3 point. | |
| | | **TOTAL POINTS** | |

Maximum possible score = 6 points. A score of ≤ 4 points represents multidimensional poverty.
